# Dynamic longitudinal discriminant analysis using multiple longitudinal markers of different types

**DOI:** 10.1177/0962280216674496

**Published:** 2016-10-26

**Authors:** David M Hughes, Arnošt Komárek, Gabriela Czanner, Marta Garcia-Fiñana

**Affiliations:** 1 Department of Biostatistics, University of Liverpool, UK; 2 Charles University, Faculty of Mathematics and Physics, Department of Probability and Mathematical Statistics, Prague, Czech Republic; 3 Department of Eye and Vision Science, University of Liverpool, UK

**Keywords:** Discriminant analysis, multivariate generalized linear mixed model, multivariate longitudinal data, random effects, mixture distributions

## Abstract

There is an emerging need in clinical research to accurately predict patients’ disease status and disease progression by optimally integrating multivariate clinical information. Clinical data are often collected over time for multiple biomarkers of different types (e.g. continuous, binary and counts). In this paper, we present a flexible and dynamic (time-dependent) discriminant analysis approach in which multiple biomarkers of various types are jointly modelled for classification purposes by the multivariate generalized linear mixed model. We propose a mixture of normal distributions for the random effects to allow additional flexibility when modelling the complex correlation between longitudinal biomarkers and to robustify the model and the classification procedure against misspecification of the random effects distribution. These longitudinal models are subsequently used in a multivariate time-dependent discriminant scheme to predict, at any time point, the probability of belonging to a particular risk group. The methodology is illustrated using clinical data from patients with epilepsy, where the aim is to identify patients who will not achieve remission of seizures within a five-year follow-up period.

## 1 Introduction

In many clinical studies, increasingly complex data are collected. The complexity of the data may be due to its multivariate and longitudinal nature as measurements are often obtained for multiple biomarkers over time. Data of this kind have a complex correlation structure with correlation, for each patient, between measurements of a biomarker at different time points and between observed values of multiple biomarkers at a single time point. An additional complication is that collected data are often of varying types, with data being potentially continuous, counts, binary, or having multiple categories. Finally, the time points at which biomarkers are measured may be different between biomarkers and between individuals for a given biomarker.

Frequent clinical interest is in being able to classify patients into various groups corresponding to severity of their disease status or disease progression, based on the evolution of biomarkers observed over time. Our goal in this paper is to present a flexible and dynamic approach in which we use available longitudinal data on multiple biomarkers of various types to accurately classify patients into groups (such as diagnosis groups) in a discriminant analysis and to do so as early as possible.

### 1.1 Clinical motivation

We consider data from a study of patients with epilepsy to motivate our developments. We are interested in being able to identify those patients who will not achieve remission from seizures within five years of commencing treatment. For the purposes of this paper, this group of patients will be referred to as the refractory group. By contrast, a patient is defined as being in remission if they have had a continuous 12-month period without any seizures at any point within five years from diagnosis. Our aim is to use multivariate longitudinal clinical data from patients with epilepsy to identify, as early as possible, if a particular patient belongs to the refractory group. Early classification would allow clinicians to try alternative treatments with the hope of achieving adequate seizure control. Consequently, patients could be spared some time on unsuitable treatment regimes and receive more effective, individualised treatment.

Data were acquired from the Standard and New Antiepileptic Drugs (SANAD) study^1,2^ which involved patients diagnosed with epilepsy between December 1999 and August 2004. Follow-up data on these patients are available up until January 2006. Here, 1772 patients from the SANAD database are considered. These patients have been followed up sufficiently long to be known to belong to either the refractory group or the remission group. For all patients, biomarkers of different type (continuous, counts and binary) were collected over time. We remark that it is indeed possible for a patient to achieve remission and then begin to have seizures again but this is not considered in our application. For simplicity, once a patient achieves remission, they are considered as belonging to the remission group and all longitudinal measurements subsequent to the visit at which remission was achieved are discarded.

Most patients had annual clinic visits, although in some cases, the visits took place more often than annually. Information about the number and type of seizures as well as adverse events the patient has experienced since the previous visit were collected. A number of baseline covariates were also collected at the commencement of treatment (based on clinical relevance), including the patient’s age and gender, epilepsy type, whether any family members had a history of seizures, whether the patient had learning or neurological difficulties and to which arm of the SANAD study the patient had been assigned.

Out of the 1772 patients investigated, 1593 patients were in the remission group and 179 patients were in the refractory group. The median (min, lower-quartile, upper-quartile, max) follow-up times (in days) in the remission group was 710 (365, 480, 863, 1821) whilst in the refractory group was 1512 (1463, 1659, 1825, 1825). The difference in medians is easily explained due to the fact that patients who achieve remission will generally be observed for less than five years (the majority achieving remission within three years), whereas refractory patients need to be observed for at least four years to determine the refractory status.

In the following, we will consider three longitudinal markers, namely whether a patient had seizures or not since their last visit, which is binary, a transformation of the total number of seizures since their last visit (using the transformation log(1+totalseizures)), which is treated as a continuous variable, and the number of adverse events experienced since the last visit.

**Figure 1.**
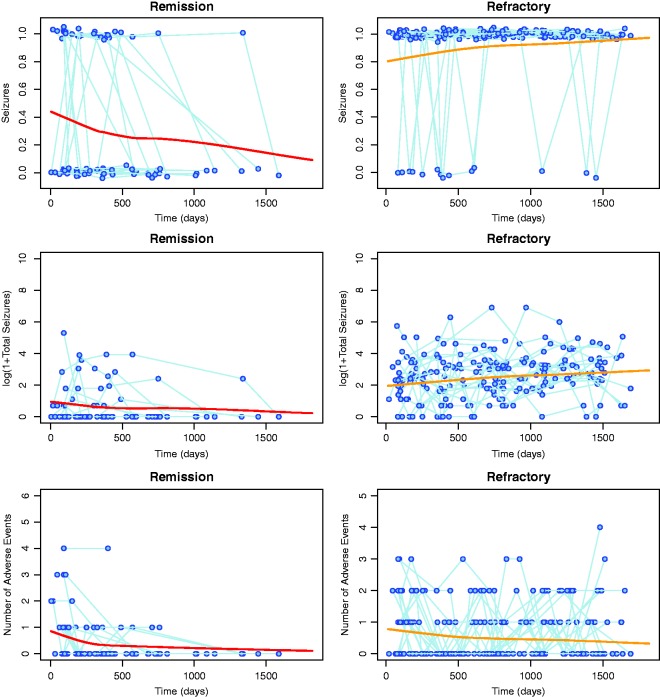
Observed longitudinal profiles of an indicator of whether a patient had seizures, log(1+total seizures) and number of adverse events experienced since the last visit for patients from the Remission group (left column) and the Refractory group (right column). In both groups, profiles of only 20 randomly selected patients are shown for clarity. Solid bold lines show LOESS smoothed profiles calculated using data from all patients. The data indicating whether a patient had seizures or not have been vertically jittered to aid interpretation.

Figure 1 shows the change over time in the levels of each of the considered biomarkers for a sample of 20 patients in each diagnostic group. As expected, in the remission group, fewer patients experience seizures since their last visit than in the refractory group. In the refractory group, the likelihood of the patient having experienced seizures since their last visit increases with time, whilst in the remission group it decreases. For patients who achieve remission, the number of seizures decreases over time, whereas for refractory patients, the number of seizures experienced remains high. It is interesting to note the most dramatic increase/decrease occurs within the first 500 days of receiving treatment. In the refractory group, the number of seizures experienced increases with time, with again, the main increase occurring during the first 500 days. The difference between the two groups for the number of adverse events experienced is less noticeable. Initially, both groups experience similar numbers of adverse events but as time increases the refractory patients appear more likely to be experiencing more adverse events than the remission patients.

In summary, Figure 1 highlights the challenges of the epilepsy data: having three longitudinal markers of different type, measured at different time points within and across subjects. The differences in remission and refractory groups can be subtle when each biomarker is considered individually. In this work, we aim to model the markers simultaneously and to use the model for discrimination between groups.

### 1.2 Dynamic longitudinal discriminant analysis

The SANAD data have been primarily analysed elsewhere,^1–4^ with most previous work concentrated on modelling of time to seizures using the baseline characteristics as prognostic factors.^3^ A different problem will be tackled in this paper. For each patient in our dataset, we have information on not only their baseline characteristics and values of the longitudinal biomarkers but also on whether they belong to the refractory or to the remission group. It is our aim to use these data to develop a statistical approach which can be used to predict the five-year seizure status (i.e., pertinence into either the refractory or the remission group) of a new patient based on their baseline characteristics as well as longitudinally gathered biomarkers. As such, the problem can be classified as a problem of longitudinal discriminant analysis (LoDA).

In addition, we aim to refine the prediction of the seizure status whenever new longitudinal observations become available at each consecutive visit. To predict the patient’s seizure status at a particular time point, we can use not only the last available longitudinal measurements (as is often the case in clinical practice) but the whole longitudinal history of relevant biomarkers known by the time we are conducting the prediction. Due to this dynamic update of the seizure status prediction, we will refer to dynamic LoDA.

To formalize our research problem, let us assume that patients are to be classified into *G* > 1 prognostic groups (*G* = 2 in the SANAD application where the prognostic groups are the refractory and the remission group). Let the group to which a patient belongs be represented by a value of the random variable U∈{0,…,G-1} which is only observable at time *T* > 0. Furthermore, suppose that information on the group membership can be predicted from R≥1 longitudinally gathered markers (*R* = 3 for the SANAD application). Let $Y_{r}=(Y_{r,1},\ldots ,Y_{r,n_{r}})$ denote a random vector representing the full longitudinal history of the *r*th marker (r=1,…,R) being observed on a particular patient at time points $t_{r}=(t_{r,1},\ldots ,t_{r,n_{r}}),t_{r,1}<\ldots <t_{r,n_{r}}<T$. Note that we do not require equal time sequences $t_{1},\ldots ,t_{R}$ for different markers, reflecting a common clinical scenario where not necessarily all markers are examined at all visits and allowing for a situation where each marker has its own visit scheme. Furthermore, let $v_{r,1},\ldots ,v_{r,n_{r}}\in {\mathbb{R}}^{p_{r}}$ be vectors of additional baseline as well as possibly time-dependent covariates that may explain evolution of the longitudinal markers $Y_{r,1},\ldots ,Y_{r,n_{r}}$ and possibly contribute to discrimination. Let

$$C=\{t_{1},\ldots ,t_{R},v_{1,1},\ldots ,v_{R,n_{R}}\}$$

denote complete information on the visit times and other covariates. For the SANAD application, apart from time, baseline covariates will be considered and will include those mentioned in Section 1.1.

For given *t* (0<t<T), let $Y_{r}(t)$ be a subvector of $Y_{r}$ covering the measurements $Y_{r,j}$ with $t_{r,j}\leq t$ ($j=1,\ldots ,n_{r}$), i.e., longitudinal measurements of the *r*th marker by time *t*. Analogously, let C(t) denote the covariate information by time *t* and finally, let $Y(t)=(Y_{1}(t),\ldots ,Y_{R}(t))$ be a random vector covering the observed values of all considered markers by time *t*.

A task of the dynamic LoDA is to use, at a given time point *t* (mostly corresponding to the visit time of a particular patient), the longitudinal history Y(t) along with the covariate information, C(t), both known by time *t*, to predict the value of the group allocation variable *U*, i.e., to predict the future prognosis of a patient by allocating them into one of *G* prognostic groups. To develop a classification procedure, it is assumed that a training (historical) dataset (the SANAD dataset in our case) is available where both the group allocations and the longitudinal measurements along with the covariate values are available.

In order to avoid misunderstanding, we point out that a similar term *dynamic prediction* is nowadays used for a problem which received considerable attention in recent years but is different from that of ours. Namely, dynamic prediction is nowadays most often referred to in the context of time-to-event analysis where it refers to estimation of a patient-specific survival distribution given their baseline and longitudinal characteristics. This estimation is then repeated in time (dynamically) as new longitudinal information becomes available. Classical methods in this context include landmarking (see the overview by van Houwelingen and Putter^5^) and usage of methods of joint modelling of longitudinal and time-to-event data.^6–9^

In contrast to those methods, we do not deal with dynamic estimation of a subject-specific time-to-event distribution. We consider dynamic discriminant analysis where we aim to use historical data to predict dynamically (also as new longitudinal information becomes available) the group membership of a patient which is only known in the future.

Finally, note that most of the longitudinal biomarkers in the SANAD data (and many other clinical applications) are either binary or counts, in which case existing methodology for LoDA is scarce and largely unsuitable as will be indicated below.

### 1.3 LoDA based on mixed models

Classical methods of discriminant analysis, see, e.g., Chapter 4 of Hastie et al.^10^ like linear discriminant analysis or discrimination based on logistic regression do not apply in our context. These methods are often applied when only baseline characteristics or other cross-sectional characteristics related to a chosen time point, common for all patients, are to be used for discrimination. In more recent years, relevant work has been done in capturing the longitudinal nature of clinical data and using it for classification via methods of LoDA.^11–18^ These authors base their LoDA methodologies on the classical linear mixed model^19^ and propose discriminant methods based on longitudinal measurements of a single (*R* = 1) continuous marker.

Nevertheless, using a single marker may be insufficient to accurately classify the subjects into prognostic groups. By using multiple markers (*R* > 1), we may be able to more accurately classify individuals using their longitudinal information. However, fewer developments have been made in the use of multiple longitudinal markers for discrimination. We can mention Marshall et al.^20^ who use several continuous markers and a multivariate non-linear mixed model to discriminate between women with and without pregnancy abnormalities. Komárek et al.^21^ use three continuous markers and a multivariate linear mixed model to evaluate a prognosis of primary biliary cirrhosis patients. In a similar way, Morrell et al.^22^ use three continuous markers to predict the presence of prostate cancer.

As indicated above, most methods of LoDA exploit mixed model methodology. A benefit of its usage is that data do not have to be measured at regular intervals. It is possible for patients to be observed different numbers of times and at irregularly spaced intervals. In addition, it is not necessary for all biomarkers to be measured on each patient at each visit. For example, it is possible for one biomarker to be measured at one visit and then another at a different visit. This flexibility is useful in clinical applications where regularly spaced observations are rarely achieved, and not all biomarkers are measured at the same time point.

Unfortunately, the above referenced methodologies are not directly suitable when there are markers that are not all continuous (as in our application). A related development made towards LoDA with multiple markers of various types has been made by Fieuws et al.^23^ who predict renal graft failure using combination of linear, non-linear and generalized linear mixed models (GLMMs). All considered markers are combined into a multivariate mixed model by specifying a joint distribution for the random effects. Computational complexity of the maximum likelihood estimation (MLE) is tackled by using a so-called pairwise fitting approach which proved to be a useful approximation towards MLE. They also show that the prediction is better when considering multiple markers than by considering only a single marker.

In LoDA methods based on multivariate longitudinal markers, the complex correlation structure between various markers is mostly taken into account by assuming a joint distribution for all random effects in the underlying mixed models. In each of the references mentioned previously, except for Komárek et al.,^21^ the random effects are assumed to follow a normal distribution. However, as shown by Verbeke and Lesaffre,^24^ this assumption cannot easily be checked. Moreover, under misspecification of the random effects distribution, estimates of the mixed model parameters may become seriously biased^25^ and consequently, the performance of the discriminant procedure may also be affected. In the mixed models literature, several extensions avoiding the normality assumption for the random effects have been proposed.^26^ Nevertheless, applications of such models in the LoDA context are still rare. One of the few works in this direction is described by Komárek et al.^21^ who consider a multivariate linear mixed model with distribution of random effects specified as a finite normal mixture, which robustifies the model towards misspecification of the random effects distribution. To overcome the computational complexity of the MLE, they use Markov Chain Monte Carlo (MCMC) methodology within a Bayesian framework.

### 1.4 Towards robust LoDA based on multivariate longitudinal markers of different types

The aim of this paper is to extend a multivariate LoDA method so that (i) it allows for multiple longitudinal markers of different types as requested by data from the SANAD study and (ii) the underlying model is robustified against possible misspecification of the random effects distribution.

We are aware of two methodologies available in the literature that satisfy either (i) or (ii) but none of them both of the requirements. The approach of Komárek et al.^21^ fulfils (ii) but only continuous markers can be used. We allow for binary and count biomarkers by replacing the underlying multivariate linear mixed model with the multivariate generalized linear mixed model (MGLMM).

On the other hand, the method of Fieuws et al.^23^ allows for markers of different nature but normality of the random effects is assumed. By using a pairwise fitting approach, these authors attempt to overcome the complexity of finding the maximum likelihood parameter estimates. In this paper we take a different approach by using Bayesian methods with MCMC estimation and considering a normal mixture in the distribution of random effects to robustify the model against misspecification of the random effects distribution.

Conceptually, the LoDA methodology proposed here follows that of Komárek et al.^21^ Nevertheless, to allow also for binary and count biomarkers, we replace the underlying multivariate linear mixed model used therein by the MGLMM. To robustify the model against misspecification of the random effects distribution, we shall consider a normal mixture in the distribution of random effects. In this paper, we obtain a robust group-specific model that will be further used in the LoDA procedure.

An outline of the remainder of the paper is as follows. In Section 2, we describe the MGLMM with a mixture distribution for the random effects. This allows us to jointly model the longitudinal profile of each marker in each prognostic group. We also describe the MCMC procedure that is applied to infer on the model parameters. Section 3 describes the LoDA used to classify new patients into prognostic groups. An example of our methodology applied to the SANAD data is shown in Section 4 with a summary provided in Section 5.

## 2 MGLMMs with a normal mixture in the random effects distribution

### 2.1 Model

The basis for the LoDA procedure, explained further in Section 3, is a MGLMM with a normal mixture in the random effects distribution. This is assumed for the longitudinal evolution of considered markers in each prognostic group. Specifically, given *U* = *g* (the group *g*, to which a patient belongs), g=0,…,G-1, we assume for observations of marker *r*: $Y_{r}$ (r=1,…,R), obtained at time points $t_{r}$ with covariate vectors (corresponding to potentially fixed effects, **x**, or random effects **z**) $v_{r,1},\ldots ,v_{r,n_{r}}$ a GLMM. To this end, it is assumed that a particular subject is characterized by values of a latent random effects vector $b=(b_{1},\ldots ,b_{R})$ and the *j*th longitudinal observation ($j=1,\ldots ,n_{r}$) of the *r*th marker is assumed to follow, given *U* = *g* and given **b**, a distribution from an exponential family (e.g., normal, Poisson, Bernoulli) with a dispersion parameter $\varphi_{r}^{g}$ and the expectation given as

(1) $$\begin{array}{c} h_{r}^{-1}\{E(Y_{r,j}|b,U=g)\}=x_{r,j}^{g⊤}\alpha_{r}^{g}+z_{r,j}^{g⊤}b_{r},r=1,\ldots ,R,j=1,\ldots ,n_{r} \end{array}$$

In (1), $h_{r}^{-1}$ is a known link function used in the GLMM for the *r*th marker (e.g., logit for Bernoulli responses, log for Poisson variables), $x_{r,j}^{g}=x_{r,j}^{g}(C)$ and $z_{r,j}^{g}=z_{r,j}^{g}(C)$ are covariate vectors used in a model for the prognostic group *g* derived from the information on the visit times and the covariates C. Note that different covariate sets **x** and **z** can be used in models for different prognostic groups. Further, $\alpha_{r}^{g}$ are unknown parameters (fixed effects) related to the model for the *r*th marker in the group *g*. As a standard feature of the exponential family, the dispersion parameter $\varphi_{r}^{g}$ is either known (e.g., being equal to 1 for Bernoulli or Poisson responses) or unknown (e.g., residual variance in a GLMM with Gaussian response).

In our SANAD example, we consider *R* = 3 longitudinal biomarkers, $Y_{1}$ denotes a vector of binary variables that represent whether or not the patient experienced seizures since the last clinic visit, $Y_{2}$ denotes a vector holding the total numbers of seizures since the last clinic visit under the transformation log(1+totalseizures) and the vector $Y_{3}$ records the numbers of adverse events experienced since the previous clinic visit. Each biomarker is modelled in each group using the same set of six covariates, i.e., $x_{r,j}^{g}=(x_{j,1},\ldots ,x_{j,6}{)}^{⊤}$, where $x_{j,1},\ldots ,x_{j,6}$ corresponds to (1) time since last visit, (2) time since diagnosis, (3) age at diagnosis, (4) epilepsy type, (5) sex and (6) randomization period. With respect to the random effects structure, the model of each marker in each group contains a random intercept. This means $z_{r,j}^{g}=1$ and a three-dimensional random effects vector $b=(b_{1},b_{2},b_{3}{)}^{⊤}$ (random intercepts for the three markers) is involved. More details on the model parameters and biomarkers are given in Section 4.

Possible correlation between repeated observations of both the same marker and different markers measured on the same patient is accounted for by inclusion of the random effect vector **b**. Given its value, all single longitudinal measurements $Y_{1,1},\ldots ,Y_{R,n_{R}}$ are assumed to be independent. Traditionally, it is assumed that the random effect vector **b** follows a normal distribution. Nevertheless, as pointed out in the introduction, this assumption is difficult to assess and may have a crucial impact on the validity of the statistical inference we aim to conduct using the proposed model. A suitable flexible model robustified towards misspecification of the random effects distribution consists of assuming a normal mixture for the random effects. For our model towards LoDA, possibly different normal mixtures should be considered in different prognostic groups. Hence, formally, we assume

(2) $$\begin{array}{c} b|U=g\;∼\sum_{k=1}^{K^{g}}w_{k}^{g}MVN(\mu_{k}^{g},D_{k}^{g}) \end{array}$$

where MVN(μ,D) stands for a multivariate normal distribution with the mean vector ***μ*** and a covariance matrix D. Unknown parameters of the mixture model (2) in the prognostic group *g* are the mixture weights $w^{g}=(w_{1}^{g},\ldots ,w_{K^{g}}^{g})$ ($0<w_{k}^{g}<1,k=1,\ldots ,K^{g},\sum_{k=1}^{K^{g}}w_{k}^{g}=1$), the mixture means $\mu_{1}^{g},\ldots ,\mu_{K^{g}}^{g}$ and the mixture covariance matrices $D_{1}^{g},\ldots ,D_{K^{g}}^{g}$. The number of mixture components, *K_g_* is initially assumed to be known. We return to its choice later in Section 4.2.

As mentioned above, a primary purpose of usage of the mixture in equation (2) is to robustify our model against misspecification of the random effects distribution. At this place, we should mention that also in other contexts, mixtures proved to provide a flexible distributional model^26–28^and hence can be considered as a sort of robustification against violation of the assumption on the random effects distribution.

In the following, let $\psi^{g}$ denote a vector of unknown parameters of the GLMM model (1) in group *g*. That is, $\psi^{g}$ consists of the fixed effects $\alpha_{1}^{g},\ldots ,\alpha_{R}^{g}$ and a subset of the dispersion parameters $\varphi_{1}^{g},\ldots ,\varphi_{R}^{g}$ that are not constant for given exponential family distribution. Analogously, let $\theta^{g}$ denote a vector of unknown parameters of the mixture model (2) in the distribution of random effects in group *g*. That is, $\theta^{g}$ consists of the mixture weights $w^{g}$, the mixture means $\mu_{1}^{g},\ldots ,\mu_{K^{g}}^{g}$ and the mixture covariance matrices $D_{1}^{g},\ldots ,D_{K^{g}}^{g}$. For observed values $y_{1}=(y_{1,1},\ldots ,y_{1,n_{1}}),\ldots ,y_{R}=(y_{R,1},\ldots ,y_{R,n_{R}})$ of the longitudinal markers $Y=(Y_{1},\ldots ,Y_{R})$ for a subject from the prognostic group *g*, an implied (marginal) density $f_{g}^{\mathrm{marg}}(\cdot ;\psi^{g},\theta^{g},C)$ is

(3) $$f_{g}^{\mathrm{marg}}(y_{1},\ldots ,y_{R};\psi^{g},\theta^{g},C)=\int f_{g}^{\mathrm{cond}}(y_{1},\ldots ,y_{R}|b;\psi^{g},C)f_{g}^{\mathrm{ranef}}(b;\theta^{g})\text{d}b$$

where $f_{g}^{\mathrm{cond}}(\cdot ;|b;\psi^{g},\theta^{g})$ denotes a (conditional) density of the observed markers given the random effect vectors and finally, $f_{g}^{\mathrm{ranef}}(\cdot ;\theta^{g})$ is a density of the random effects. For the multivariate GLMM with a normal mixture in the random effects distribution, we have

(4) $$f_{g}^{\mathrm{cond}}(y_{1},\ldots ,y_{R}|b;\psi^{g},C)=\overset{R}{\underset{r=1}{\Pi }}\overset{n_{r}}{\underset{j=1}{\Pi }}p_{r}(y_{r,j}|b;\psi^{g},C)$$

(5) $$\begin{array}{c} f_{g}^{\mathrm{ranef}}(b;\theta^{g})=\sum_{k=1}^{K^{g}}w_{k}^{g}\phi (b;\mu_{k}^{g},D_{k}^{g}) \end{array}$$

where $p_{r}(\cdot |b;\psi^{g},C)$ is a density of the exponential family distribution assumed for the *r*th marker, r=1,…,R, whose expectation depends on the random effects vector **b**, the fixed effects $\alpha_{r}^{g}$ (subvector of the parameter vector $\psi^{g}$) and on the covariate information C by the GLMM model (1). Further, ϕ(·;μ,D) denotes a density of the multivariate normal distribution with mean ***μ*** and a covariance matrix D.

### 2.2 Sampling-based Bayesian inference

For a training dataset of size *N*, composed of observed values $y_{i,1}=(y_{i,1,1},\ldots ,y_{i,1,n_{i,1}}),\ldots ,y_{i,R}=(y_{i,R,1},\ldots ,y_{i,R,n_{i,R}})$ of the longitudinal markers $Y_{i}=(Y_{i,1},\ldots ,Y_{i,R})$, component allocations *U_i_* = *u_i_*, the visit times $t_{i,r}=(t_{i,r,1},\ldots ,t_{i,r,n_{i,r}})$ and the covariate vectors $v_{i,r,j},i=1,\ldots ,N,r=1,\ldots ,R,j=1,\ldots ,n_{i,r}$, a likelihood basing the inference on the model parameters for a prognostic group g∈{0,…,G-1} is (while assuming independence between the study subjects)

(6) $$\begin{array}{c} L_{g}(\psi^{g},\theta^{g})=\underset{i:u_{i}=g}{\Pi }f_{g}^{\mathrm{marg}}(y_{i,1},\ldots ,y_{i,R};\psi^{g},\theta^{g},C_{i})\\ =\underset{i:u_{i}=g}{\Pi }\int f_{g}^{\mathrm{cond}}(y_{i,1},\ldots ,y_{i,R}|b_{i};\psi^{g},C_{i})f_{g}^{\mathrm{ranef}}(b_{i};\theta^{g})\text{d}b_{i}\\ =\underset{i:u_{i}=g}{\Pi }\int \overset{R}{\underset{r=1}{\Pi }}\overset{n_{i,r}}{\underset{j=1}{\Pi }}p_{r}(y_{i,r,j}|b_{i};\psi^{g},C_{i})\{\sum_{k=1}^{K^{g}}w_{k}^{g}\phi (b_{i};\mu_{k}^{g},D_{k}^{g})\}\text{d}b_{i}, \end{array}$$

where $C_{i}=\{t_{i,1},\ldots ,t_{i,R},v_{i,1,1},\ldots ,v_{i,R,n_{i,R}}\},i=1,\ldots ,N$. Note that (6) could also be written as

$$L_{g}(\psi^{g},\theta^{g})=\underset{i:u_{i}=g}{\Pi }\{\sum_{k=1}^{K^{g}}w_{k}^{g}\int \overset{R}{\underset{r=1}{\Pi }}\overset{n_{i,r}}{\underset{j=1}{\Pi }}p_{r}(y_{i,r,j}|b_{i};\psi^{g},C_{i})\phi (b_{i};\mu_{k}^{g},D_{k}^{g})\text{d}b_{i}\}$$

and hence the MGLMM with a normal mixture in the random effects distribution that we use to model a longitudinal evolution of the markers in each of the prognostic groups, can also be interpreted as a mixture of the MGLMM’s with a normal distribution of random effects. This allowed Komárek and Komárková^29^ to use the model for clustering (i.e., unsupervised classification) based on longitudinal data. Use of their clustering methodology in our context would mean (unsupervised) division of subjects of a given *g*th prognostic group into additional smaller subgroups which is not the aim of this paper. Nevertheless, we can exploit a methodology developed in Komárek and Komárková^29^ for estimation of unknown parameters $\psi^{g}$ and $\theta^{g}$ for each prognostic group g∈{0,…,G-1}.

Due to a mixture nature of the likelihood (6) which additionally involves analytically intractable integration where the integrand combines a general exponential family and a normal density, maximum-likelihood-based inference is tractable only with difficulties. For this reason, a MCMC-based Bayesian estimation as proposed by Komárek and Komárková^29^ will be adopted here. In the following, let *p* be a generic symbol for a density. Bayesian inference in the prognostic group g∈{0,…,G-1} consists of specifying a prior distribution $p(\psi^{g},\theta^{g})$ for the model parameters and then basing the inference on the posterior distribution $p(\psi^{g},\theta^{g}|Y_{g})$, where $Y_{g}=\{Y_{i}:u_{i}=g\}\subset Y=\{Y_{i}:i=1,\ldots ,N\}$ represent observed longitudinal markers in group *g*. Using Bayes theorem, the posterior distribution combines the prior distribution and the likelihood (6) as

(7) $$p(\psi^{g},\theta^{g}|Y_{g})\propto L_{g}(\psi^{g},\theta^{g})p(\psi^{g},\theta^{g})$$

Komárek and Komárková^29^ describe (i) how to specify the prior distribution $p(\psi^{g},\theta^{g})$ in a weakly informative way if no prior information on the model parameters is available, (ii) how to use the MCMC methodology to obtain a sample

$$\begin{array}{c} S_{g}=\{(\psi^{g,(m)},\theta^{g,(m)}):m=1,\ldots ,M\},\\ \psi^{g,(m)}=(\alpha_{1}^{g,(m)},\ldots ,\alpha_{R}^{g,(m)},\varphi_{1}^{g,(m)},\ldots ,\varphi_{R}^{g,(m)}),\\ \theta^{g,(m)}=(w_{1}^{g,(m)},\ldots ,w_{K^{g}}^{g,(m)},\mu_{1}^{g,(m)},\ldots ,\mu_{K^{g}}^{g,(m)},D_{1}^{g,(m)},\ldots ,D_{K^{g}}^{g,(m)}) \end{array}$$

of size *M* from the posterior distribution (7), (iii) how to infer on a number of mixture components *K_g_* in a mixture distribution (5) assumed for random effects. We refer therein for details. Moreover, an implementation of the MCMC methodology is available as a contributed package mixAK^30^ of the R software.^31^

Finally, if it is assumed that the model parameters for different prognostic groups are apriori independent and a *joint* prior distribution for model parameters $\psi =(\psi^{0},\ldots ,\psi^{G-1}),\theta =(\theta^{0},\ldots ,\theta^{G-1})$ from all prognostic groups takes a product form $p(\psi ,\theta )=\overset{G-1}{\underset{g=0}{\Pi }}p(\psi^{g},\theta^{g})$, a sample $S=\{S_{0},\ldots ,S_{G-1}\}$ obtained by combining *G* independently obtained samples $S_{0},\ldots ,S_{G-1}$ is then also a sample from the *joint* posterior distribution p(ψ,θ|Y) of the model parameters for all prognostic groups given the full training dataset Y. This follows from a classical assumption of independence between the study subjects which gives a product form of the likelihood of the full training dataset being $L(\psi ,\theta )=\overset{G-1}{\underset{g=0}{\Pi }}L_{g}(\psi^{g},\theta^{g})$ leading to the product form of the posterior distribution

(8) $$p(\psi ,\theta |Y)=\overset{G-1}{\underset{g=0}{\Pi }}p(\psi^{g},\theta^{g}|Y_{g})$$

## 3 LoDA procedure

Let $Y_{\mathrm{new}}=(Y_{\mathrm{new},1},\ldots ,Y_{\mathrm{new},R})$ denote a random vector that represents observed values $y_{\mathrm{new},1},\ldots ,y_{\mathrm{new},R}$ of the longitudinal markers for a new subject (in general known by some time *t* < *T* but we suppress this in notation for clarity) that is to be classified into one of the *G* prognostic groups and let $C_{\mathrm{new}}=\{t_{\mathrm{new},1},\ldots ,t_{\mathrm{new},R},v_{\mathrm{new},1,1},\ldots ,v_{\mathrm{new},R,n_{\mathrm{new},R}}\}$ be the corresponding visit times and other covariate values (again, possibly known by some time *t* < *T*). Further, let $U_{\mathrm{new}}\in \{0,\ldots ,G-1\}$ be a random variable that represents allocation of the new subject into one of the *G* groups. At this point, we assume that a value *u_new_* of *U_new_* is not observed and it is our aim to predict it using the LoDA procedure based on the training dataset. Before we do so, additional notation is needed. Let $\pi_{g}=P(U_{\mathrm{new}}=g),g=0,\ldots ,G-1$ denote prevalences of the prognostic groups in the study population ($0<\pi_{g}<1,g=0,\ldots ,G-1,\sum_{g=0}^{G-1}\pi_{g}=1$) which, as is common in applications of the discriminant analysis, are assumed to be known in advance and are often called in this context *prior* group probabilities.

### 3.1 Full Bayesian prediction

Having proposed the Bayesian inference for the model parameters using the training dataset Y, the problem of classification of a new subject in a full Bayesian setting coincides with a problem of estimating posterior probabilities

$$P_{\mathrm{new},g}^{\mathrm{marg}}=P(U_{\mathrm{new}}=g|Y_{\mathrm{new}},Y),g=0,\ldots ,G-1$$

Here, $Y_{\mathrm{new}}$ denotes the longitudinal information for a new patient. Specifically, in the context of the SANAD study, it denotes all the longitudinal information available for a patient up until the time at which a prediction is to be made. It then follows from decision theory for classification^10^ that if costs of all types of misclassification are the same, the new subject is classified into that group for which $P_{\mathrm{new},g}^{\mathrm{marg}}$ is maximal. That is, U^new=u^new, such that Pnew,u^newmarg=maxg=0,…,G-1Pnew,gmarg. Different strategies can, however, be adopted on how to exploit the posterior group allocation probabilities towards classification depending on a clinical importance of different types of misclassification, see Section 4.1 for illustration.

To calculate $P_{\mathrm{new},g}^{\mathrm{marg}}$, we first note that

(9) $$\begin{array}{c} P_{\mathrm{new},g}^{\mathrm{marg}}=\int P(U_{\mathrm{new}}=g|Y_{\mathrm{new}},\psi ,\theta ,Y)p(\psi ,\theta |Y)\text{d}(\psi ,\theta )\\ =E_{p(\psi ,\theta |Y)}P(U_{\mathrm{new}}=g|Y_{\mathrm{new}},\psi ,\theta ,Y),g=0,\ldots ,G-1 \end{array}$$

where $E_{p(\psi ,\theta |Y)}$ denotes expectation with respect to the posterior distribution (8) of the model parameters given the training dataset. If it is further assumed, as is common in this setting, that given the knowledge of the model parameters, a training dataset Y does not bear any additional information concerning the new subject, we obtain (for g=0,…,G-1)

(10) $$P(U_{\mathrm{new}}=g|Y_{\mathrm{new}},\psi ,\theta ,Y)=P(U_{\mathrm{new}}=g|Y_{\mathrm{new}},\psi ,\theta )=:P_{\mathrm{new},g}^{\mathrm{marg}}(\psi ,\theta )$$

where another use of Bayes theorem provides

$$\begin{array}{c} P_{\mathrm{new},g}^{\mathrm{marg}}(\psi ,\theta )=\frac{\pi_{g}f_{g}^{\mathrm{marg}}(y_{\mathrm{new},1},\ldots ,y_{\mathrm{new},R};\psi^{g},\theta^{g},C_{\mathrm{new}})}{\sum_{\overset{∼}{g}=0}^{G-1}\pi_{\overset{∼}{g}}f_{\overset{∼}{g}}^{\mathrm{marg}}(y_{\mathrm{new},1},\ldots ,y_{\mathrm{new},R};\psi^{\overset{∼}{g}},\theta^{\overset{∼}{g}},C_{\mathrm{new}})} \end{array}$$

With the frequentist (non-Bayesian) LoDA methodologies,^32,33^ classification of the new subjects is usually based on the group probabilities (10), in which the unknown parameters $\psi^{g},\theta^{g},g=1,\ldots ,G$ are replaced by their suitable estimates, e.g., maximum-likelihood estimates. On the other hand, the full Bayesian approach dictates to use the posterior probabilities $P_{\mathrm{new},g}^{\mathrm{marg}}$ (9), which are the posterior means (over the posterior distribution of the unknown parameters) of the group probabilities $P_{\mathrm{new},g}^{\mathrm{marg}}(\psi ,\theta )$ (10). Having used the MCMC inference, the values of $P_{\mathrm{new},g}^{\mathrm{marg}}$ are approximated using the generated samples $S_{0},\ldots ,S_{G-1}$ as

(11) P^new,gmarg=1M∑m=1MPnew,gmarg(ψ(m),θ(m)),g=0,…,G-1

Finally, we note that when evaluating (11), analytically intractable integral from (3) is in general involved in calculation of the marginal densities $f_{g}^{\mathrm{marg}}$ (g=0,…,G-1). Komárek and Komárková^29^ use a Laplace approximation to this end and we will exploit it here as well.

### 3.2 Marginal, conditional and random effects prediction

In several previous works on LoDA based on the mixed models,^21,32,33^ the authors distinguish so-called *marginal*, *conditional* and *random effects* prediction, each having its own pros and cons and more importantly, providing prediction of different quality depending on problem at hand. Hence, in any application of the LoDA based on the mixed model, it is useful to consider all these types and then to choose that one providing the best classification results.

The *marginal* prediction in the original terminology of Morrell et al.^32^ corresponds, in fact, to using the group probabilities (10) as a basis for classification of the new subject, which next to the model parameters depend only on the values of the (observable) longitudinal markers $Y_{\mathrm{new}}=(y_{\mathrm{new},1},\ldots ,y_{\mathrm{new},R})$ of the new subject. On the other hand, for both the *conditional* and the *random effects* prediction, it is necessary to represent the new object also by the values of the (unobservable) random effect vector $b_{\mathrm{new}}$ for which the assumed joint distribution, given the group allocation, follows from the assumed models (4) and (5). That is, the joint distribution of $Y_{\mathrm{new}},b_{\mathrm{new}}$ given $U_{\mathrm{new}}=g$ has, for g=0,…,G-1, a density

(12) $$\begin{array}{c} f_{g}^{\mathrm{joint}}(y_{\mathrm{new},1},\ldots ,y_{\mathrm{new},R},b_{\mathrm{new}}|\psi^{g},\theta^{g},C_{\mathrm{new}})\\ =f_{g}^{\mathrm{cond}}(y_{\mathrm{new},1},\ldots ,y_{\mathrm{new},R}|b_{\mathrm{new}};\psi^{g},C_{\mathrm{new}})f_{g}^{\mathrm{ranef}}(b_{\mathrm{new}};\theta^{g}) \end{array}$$

where $f_{g}^{\mathrm{cond}}$ and $f_{g}^{\mathrm{ranef}}$ are given by (4) and (5), respectively.

To calculate the *random effects* prediction, the group probabilities (10) are, for g=0,…,G-1, replaced by

$$\begin{array}{c} P_{\mathrm{new},g}^{\mathrm{ranef}}(b_{\mathrm{new}}^{0},\ldots ,b_{\mathrm{new}}^{G-1},\psi ,\theta ):=\frac{\pi_{g}f_{g}^{\mathrm{ranef}}(b_{\mathrm{new}}^{g};\theta^{g})}{\sum_{\overset{∼}{g}=0}^{G-1}\pi_{\overset{∼}{g}}f_{\overset{∼}{g}}^{\mathrm{ranef}}(b_{\mathrm{new}}^{\overset{∼}{g}};\theta^{\overset{∼}{g}})} \end{array}$$

where $b_{\mathrm{new}}^{g},g=0,\ldots ,G-1$ is a suitable characteristic of the (predictive) distribution of $b_{\mathrm{new}}$ given $U_{\mathrm{new}}=g$, given the observed value of the longitudinal markers $Y_{\mathrm{new}}=(y_{\mathrm{new},1},\ldots ,y_{\mathrm{new},R})$ and given the model parameters $\psi^{g}$ and $\theta^{g}$ from the model in group *g*. This predictive distribution follows directly from the joint distribution (12)

(13) $$\begin{array}{c} p(b_{\mathrm{new}}|U_{\mathrm{new}}=g,y_{\mathrm{new},1},\ldots ,y_{\mathrm{new},R},\psi^{g},\theta^{g})\\ \propto f_{g}^{\mathrm{joint}}(y_{\mathrm{new},1},\ldots ,y_{\mathrm{new},R},b_{\mathrm{new}}|\psi^{g},\theta^{g},C_{\mathrm{new}}) \end{array}$$

The mean of this distribution, which is, in fact, the empirical Bayes estimator of the random effect value given the group is usually exploited in the LoDA procedure.^33^ With the Bayesian approach, it is natural to consider, in the mood of the Bayesian data augmentation,^34^ the unobservable random effect value $b_{\mathrm{new}}$ as additional model parameter with the prior distribution (conditioned by the allocation in group *g*) given by (4). For classification, the MCMC-based estimators

P^new,granef=1M∑m=1MPnew,granef(bnew0,(m),…,bnewG-1,(m),ψ(m),θ(m)),g=0,…,G-1

are used, where $b_{\mathrm{new}}^{g,(m)},g=0,\ldots ,G-1,m=1,\ldots ,M$ is sampled from the predictive distribution (13) with $\psi^{g}=\psi^{g,(m)}$ and $\theta^{g}=\theta^{g,(m)},m=1,\ldots ,M$.

In a similar way, the *conditional* prediction is obtained. It first replaces the group probabilities (10) by (g=0,…,G-1)

$$P_{\mathrm{new},g}^{\mathrm{cond}}(b_{\mathrm{new}}^{0},\ldots ,b_{\mathrm{new}}^{G-1},\psi ,\theta ):=\frac{\pi_{g}f_{g}^{\mathrm{cond}}(y_{\mathrm{new},1},\ldots ,y_{\mathrm{new},R}|b_{\mathrm{new}}^{g};\psi^{g})}{\sum_{\overset{∼}{g}=0}^{G-1}\pi_{\overset{∼}{g}}f_{\overset{∼}{g}}^{\mathrm{cond}}(y_{\mathrm{new},1},\ldots ,y_{\mathrm{new},R}|b_{\mathrm{new}}^{\overset{∼}{g}};\psi^{\overset{∼}{g}})}$$

With the MCMC-based Bayesian inference, the estimators

P^new,gcond=1M∑m=1MPnew,gcond(bnew0,(m),…,bnewG-1,(m),ψ(m),θ(m)),g=0,…,G-1

of the group probabilities are used for classification.

We have described here three possible methods of prediction. It is entirely possible that different choices of method would result in different predicted group status for a particular patient. In the process of testing and building the model, one must assess the predictive ability of any of the three methods to determine which works best.

## 4 Application to SANAD data

Section 1 gives an overview of the SANAD data and summary information. In this section, we present the results of the methodology presented in Sections 2 and 3 when applied to the SANAD data.

As described in Section 1, we consider three longitudinal markers to predict refractory or remission patients. For the binary marker, whether a patient had seizures or not since their last visit, we use a logistic model as the form of the GLMM. For the number of adverse events (count marker), we consider a log-Poisson model. Finally, for the number of seizures experienced since the previous visit, we utilize a log transformation of the form, log(1+totalseizures) and select a Gaussian model. These models are combined through the inclusion of jointly distributed random effects to induce correlation. We allow each longitudinal marker to have a random intercept and allow these three random intercepts to be correlated.

As explanatory fixed effect covariates, we will use (in both prognostic groups) (1) time since last visit (TLFU) in order to account for the fact the visit schedule is irregular and hence the biomarkers are not collected over a fixed time period, (2) time since diagnosis (TDiag), (3) age at *t* = 0 (Age), (4) epilepsy type (Type), a binary indicator as to whether the patient has generalized epilepsy or not, (5) sex (Sex) and (6) a binary covariate indicating whether or not recruitment occurred before 6 June 2001 (RecP). The reason for this final covariate is that a new drug was added to the trial on this date which may have introduced differences among patients in the longitudinal profiles. In the remission and the refractory prognostic group, there are 57.6% and 53.6% of males, respectively. The median (min, lower-quartile, upper-quartile, max) age at *t* = 0 in the two groups is 30 (5, 17, 47, 86) and 32 (5, 20, 42, 71), respectively. In total, 23.7% of patients in the remission group had generalized epilepsy, compared to 14% of patients in the refractory group. In the example presented here, we consider for simplicity, the case where the number of mixture components in the random effects distribution (2) is the same for each prognostic group ($K^{0}=K^{1}=K$) although this is not a necessary requirement of our methodology.

### 4.1 Dynamic LoDA procedure

As indicated in Section 1.2, we update the probabilities of a future patient’s group membership each time new information is available. This is achieved by applying repeatedly the formulas of Section 3 while taking information available by each visit time in place of $Y_{\mathrm{new}}$ and $C_{\mathrm{new}}$. In order to then use these probabilities to allocate the patient into either the refractory or remission group, we propose a dynamic discriminant analysis allocation scheme, following closely the procedure described in Brant et al.^12,22^ In our application, primary interest lies in early and correct diagnosis of refractory patients. With our dynamic LoDA procedure, we decide at each visit whether a patient can be ultimately classified as refractory or whether it is necessary to continue with their follow up before final classification can be deduced.

We proceed as follows. We consider the first clinic visit for each patient. If the estimated probability of being in the refractory group is greater than a chosen cutoff, *c*, then we assign this patient to the refractory group and stop predicting for this patient. If the probability is lower than *c*, then we proceed to the next visit and the patient remains under observation, repeating the process until either the patient has been classed as refractory or all their visits have been used. Any patient not predicted as refractory remains under observation until the last visit before their status is confirmed (either by achieving remission or by the five years since diagnosis ending). Any patient not predicted as refractory by this final visit is predicted as remission.

Of course, other schemes would have been possible. If it was the case that we were equally interested in both remission and refractory patients, we could assign a patient to either group if their probability of belonging to the group was greater than *c*, and only continue observing if neither probability was greater than *c*.

For either scheme, the cutoff *c* must be chosen by the investigator. Many methods exist to do this, depending on the needs of the investigation. We remark that with our proposed scheme, even if we classify patients into the two groups dynamically in time, only one decision concerning the group membership is taken for each patient. Consequently, classical methods of evaluation of the predictive accuracy of a binary classifier like those based on the Receiver Operating Characteristic (ROC) curve can be considered. In this paper, we select the cutoff linked to the top left most point on the ROC curve. Other alternatives, such as the Youden index, or specifying a desired sensitivity, specificity or probability of correct classification (PCC) could also be chosen. In the model building and testing stage of an analysis, a range of cutoff values can be tested and predictive accuracy compared. Following this procedure, the best cut-off can be selected and used for future classification of new patients.

In the following analysis, we use 70% of our data to train the models and the remaining 30% to test the predictive accuracy. We repeat this process 100 times in a cross-validation procedure. For each split of the data into training and test sets, we calculate various measures of predictive accuracy and average them across the 100 splits.

### 4.2 Selecting the number of components in the mixture distribution

The MGLMM introduced in Section 2 which forms the basis of the discrimination procedure considers a normal mixture (2) in the random effects distribution. In general, the number of mixture components for each of the prognostic groups, *K*^0^ and *K*^1^, must be estimated from the training dataset. Komárek and Komárková^29^ suggest to use the penalized expected deviance (PED^35^) to this end and we can, in principle, use this approach as well, separately for models in each prognostic group.

Table 1, which shows the PED values (lower value means a better model) for models with different values of *K* in the two groups, suggests to use *K* = 1 in the refractory group (although improvement on *K* = 2 is minimal). In the remission group, *K* = 3 seems to provide the best model, nevertheless, the PED improvement compared to *K* = 2 is relatively small. Note that the PED values were explored using the full data in each group before any splits of the data into training and test groups.

**Table 1.** Penalized expected deviance for models with K=1,2,3,4 mixture components in the random effects distribution.

Group	*K* = 1	*K* = 2	*K* = 3	*K* = 4
Remission	37,305	36,669	**36,578**	36,607
Refractory	**9,734**	9,740	10,403	10,497

These values were based upon the full data available in each group. The models with the best PED values are shown in bold for each group.

Nevertheless, since our primary interest lies in the prediction of the patient’s status (at a pre-specified future time point), it is more natural in our context, to evaluate the models and to select an optimal value of *K* for the random effects distribution in (2) in each group by comparing the predictive ability of each of the models using our dynamic LoDA scheme. This has been done here using the cross-validation procedure where we split the data into training and test sets 100 times and averaged the results. For simplicity, we have assumed the same number of mixture components in both the remission and refractory groups, i.e., $K^{0}=K^{1}=K$. For the sake of space, we just present here the results when using the marginal prediction method since this was the method that most consistently gave the best classification results, although the results for the conditional predictions were very similar. Table 2 shows that there is a slight improvement in specificity, positive predictive value (PPV), PCC and area under curve (AUC) when using more than one component in the mixture distribution, and particularly for *K* = 2. The other accuracy measures appear to be very similar across all considered values of *K*, although *K* = 2 shows consistently the highest value (negative predictive value). The cutoff values reported represent the choice of cutoff that gave the point on the ROC curves closest to the top left corner for each choice of *K*. The combined results from Tables 1 and 2 show that there is a benefit in using *K* > 1 mixture components in terms of PED for the remission group (with negligible loss in the refractory group), and that the classification when using these models is the same as, or slightly better than when using models with *K* = 1.

**Table 2.** Comparison of the choice of *K* and its effect on the marginal prediction accuracy.

	Cutoff	Sensitivity	Specificity	PCC	AUC	PPV	NPV
*K* = 1	0.75	0.94	0.91	0.91	0.96	0.55	0.99
*K* = 2	0.74	0.94	0.92	0.92	0.97	0.58	0.99
*K* = 3	0.67	0.93	0.91	0.91	0.96	0.56	0.99
*K* = 4	0.71	0.93	0.91	0.91	0.96	0.55	0.99

PCC: probability of correct classification; AUC: area under curve; PPV: positive predictive value; NPV: negative predictive value.

The predictions are based on 100 splits of the data where 70% of the patients in each group were used to train the MGLMMs and the remaining 30% were used to test the predictive accuracy.

### 4.3 Results of the dynamic LoDA

Having shown that there is an advantage to selecting *K* > 1 components in the distribution of the random effects, we use *K* = 2 since this gives the best classification accuracy.

A summary of the model parameter estimates is given in Table 3. The model parameters, in both the seizures and the number of seizures models, for time since recruitment switch signs between groups, which indicates that the probability of experiencing seizures and of the number of seizures experienced increases with time in the refractory group, whilst in the remission group this probability decreases. Similarly, the expected value of the random intercept for the seizures is −0.32 in the remission group and 1.25 in the refractory group. This is due to the fact that the average probability of having seizures soon after recruitment is below 0.5 in the remission group, but above 0.5 in the refractory group, which is supported by the profile plots in Figure 1.

**Table 3.** Posterior summary statistics and highest posterior density (HPD) credible intervals for the fixed effects, and random effects in a model with *K* = 2. These statistics are based on the full longitudinal data available in each group.

	Remission		Refractory	
	Posterior mean	95% HPD interval	Posterior mean	95% HPD interval
Seizures (*Y*_1_)				
TLFU (days) ($\alpha_{1,1}$)	1.2$\times 10^{-4}$	(−7.2, 9.4)$\times 10^{-4}$	1$\times 10^{-2}$	(1, 1.4)$\times 10^{-2}$
TDiag (days) ($\alpha_{1,2}$)	−3.7$\times 10^{-3}$	(−4.1, −3.3)$\times 10^{-3}$	4.8$\times 10^{-4}$	(4, 91)$\times 10^{-5}$
Age ($\alpha_{1,3}$)	−6.2$\times 10^{-3}$	(−12, 0)$\times 10^{-3}$	7.9$\times 10^{-3}$	(−1.1, 2.6)$\times 10^{-2}$
Type ($\alpha_{1,4}$)	0.64	(0.39, 0.87)	1.00	(0.30, 1.66)
Sex ($\alpha_{1,5}$)	−0.10	(−0.31, 0.12)	−0.09	(−0.69, 0.53)
RecP ($\alpha_{1,6}$)	−0.47	(−0.69, −0.26)	1.44	(−0.71, 3.75)
E(Intercept) ($\text{E}[b_{1}])$	−0.32	(−0.64, −0.01)	1.25	(−0.66, 3.83)
SD(Intercept) (SD$\left[b_{1}]\right)$	2.11	(1.94, 2.28)	3.43	(1.14, 7.62)
log(1+ number of seizures) (*Y*_2_)				
TLFU (days)($\alpha_{2,1}$)	1.1$\times 10^{-3}$	(9,14)$\times 10^{-4}$	3$\times 10^{-3}$	(2.6, 3.7)$\times 10^{-3}$
TDiag (days) ($\alpha_{2,2}$)	−1.6$\times 10^{-3}$	(−1.7, −1.5)$\times 10^{-3}$	1.6$\times 10^{-4}$	(3.8, 29)$\times 10^{-5}$
Age ($\alpha_{2,3}$)	−3.6$\times 10^{-3}$	(−5.4, −2)$\times 10^{-3}$	−5.6$\times 10^{-3}$	(−17, 4.6)$\times 10^{-3}$
Type ($\alpha_{2,4}$)	0.11	(0.03, 0.18)	0.37	(0.02, 0.77)
Sex ($\alpha_{2,5}$)	−0.06	(−0.13, 0.01)	−0.29	(−0.63, 0.04)
RecP ($\alpha_{2,6}$)	−0.11	(−0.18, −0.04)	0.41	(−0.63, 1.36)
E(Intercept) ($\text{E}[b_{2}])$	1.05	(0.96, 1.15)	1.77	(1.34, 2.22)
SD(Intercept) (SD$\left[b_{2}]\right)$	0.78	(0.74, 0.83)	1.05	(0.92, 1.20)
SD(error) $\varphi_{2}$	0.89	(0.88, 0.91)	1.11	(1.07, 1.15)
Number of adverse events (*Y*_3_)				
TLFU (days) ($\alpha_{3,1}$)	−1.3$\times 10^{-3}$	(−1.9, −1)$\times 10^{-3}$	−1$\times 10^{-3}$	(−1.6, 0)$\times 10^{-3}$
TDiag (days) ($\alpha_{3,2}$)	−1.2$\times 10^{-3}$	(−1.4, −1)$\times 10^{-3}$	−3.6$\times 10^{-3}$	(−5.3, −1.9)$\times 10^{-4}$
Age ($\alpha_{3,3}$)	7.1$\times 10^{-3}$	(3.7, 10)$\times 10^{-3}$	1.8$\times 10^{-2}$	(1, 2.6)$\times 10^{-2}$
Type ($\alpha_{3,4}$)	0.23	(0.09, 0.36)	−0.16	(−0.48, 0.16)
Sex ($\alpha_{3,5}$)	−0.09	(−0.22, 0.03)	−0.16	(−0.42, 0.11)
RecP ($\alpha_{3,6}$)	−0.28	(−0.40, −0.16)	0.63	(−0.26, 1.51)
E(Intercept) ($\text{E}[b_{3}])$	−0.90	(−1.08, −0.71)	−0.92	(−1.32, −0.53)
SD(Intercept) (SD$\left[b_{3}]\right)$	0.93	(0.84, 1.01)	0.76	(0.51, 1.16)

SD: standard deviation; TLFU: Time since Last Follow Up.

These statistics are based on the full longitudinal data available in each group.

By comparing the parameter estimates in Table 3, we can see that for patients who will ultimately achieve remission, older patients, male patients and patients without generalized epilepsy are less likely to have seizures and expected to have fewer seizures than young patients, female patients and patients with generalized epilepsy, respectively. Patients in both models are expected to experience fewer adverse events as time from diagnosis increases, perhaps because the clinicians have had more time to find suitable medication to avoid side effects in some patients.

**Figure 2.**
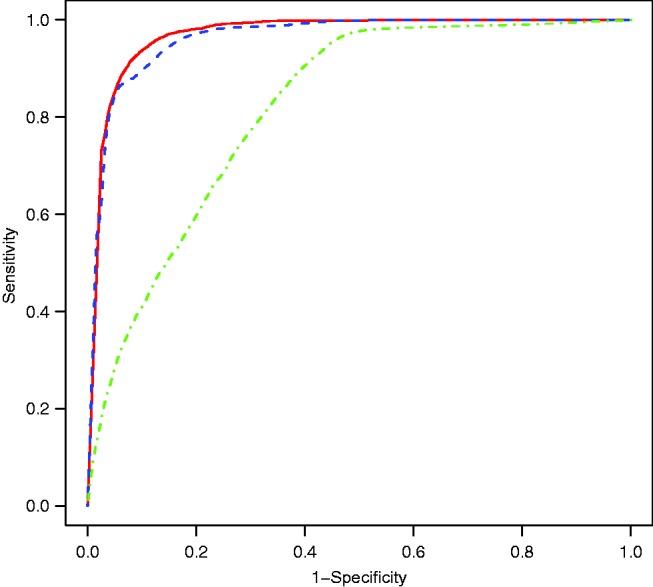
Receiver Operating Characteristic curves of the dynamic LoDA using the marginal (solid red), conditional (dashed blue) and random effects (dot dashed green) prediction methods.

The marginal and conditional dynamic LoDA approaches give good classification, as shown by high sensitivity, specificity and PCC values (see Table 5, first two columns). The random effects prediction approach works less well in this case. We are not the first to have noticed differences in the predictive accuracy of the three approaches. Komárek et al.^21^ found that the random effects prediction was the best when considering a study of primary biliary cirrhosis, whilst Morrell et al.^33^ found that the marginal method was the most successful at identifying prostate cancer patients. Which dynamic LoDA method works best seems to depend upon the application considered. The cutoff value regarded as optimal (e.g. 0.74 for the marginal prediction in Table 5) corresponds to the point closest to the top left hand corner of the ROC curve (see Figure 2). We point out here that the three methods of prediction are to be regarded as alternative competing potential classifiers. As such, there is no reason to expect that they give similar performance. Each method has a different cutoff that is optimal for that method. This is to be expected, and we note that these cutoffs are not directly comparable, since the probabilities they relate to are not the same.

**Table 4.** The longitudinal observations on a randomly selected refractory and remission patient.

time	*P*(*refractory*)	Seizures	Total number of seizures	Number of adverse events
Patient (a)				
93	0.15	Yes	10	3
184	0.21	Yes	36	2
366	0.33	Yes	40	0
720	0.71	Yes	70	0
833	**0.91**	Yes	30	3
924	0.99	Yes	100	3
1101	1	Yes	150	0
1295	1	Yes	72	0
1480	1	Yes	100	0
Patient (b)				
84	0.02	No	0	1
259	0.00	No	0	0
418	0.23	Yes	20	0
509	0.12	No	0	2
718	0.02	No	0	0
862	–	No	0	2

The refractory patient was a 35-year-old male with generalized epilepsy randomized before 6 June 2001, whilst the remission patient was a 44-year-old male with generalized epilepsy also randomized before 6 June 2001.

**Table 5.** Summary of the classification accuracy for each of the marginal, conditional and random effects methods and for traditional LDA and QDA.

	Marginal	Conditional	Random effects	Marginal (full data)	Conditional (full data)	Random effects (full data)	LDA	QDA
Cutoff	0.74	0.44	0.27	0.52	0.22	0.16	0.17	0.33
Sensitivity	0.94	0.91	0.82	0.93	0.93	0.84	0.80	0.80
Specificity	0.92	0.91	0.72	0.94	0.92	0.80	0.74	0.74
PCC	0.92	0.91	0.73	0.94	0.92	0.80	0.74	0.75
AUC	0.97	0.96	0.83	0.96	0.95	0.89	0.76	0.48
PPV	0.59	0.56	0.26	0.65	0.59	0.34	0.26	0.27
NPV	0.99	0.99	0.97	0.99	0.99	0.98	0.97	0.97
Mean lead time (days)	651	634	1000	75	78	65		
Mean prediction time (days)	876	899	522	1450	1451	1459		

LDA: linear discriminant analysis; QDA: quadratic discriminant analysis; PCC: probability of correct classification; AUC: area under curve; PPV: positive predictive value; NPV: negative predictive value.

These results are based on averages across 100 splits of the data into training and test sets. For the dynamic LoDA (first three columns), prediction stops if a patient is predicted as refractory whilst for full data predictions (columns 4 to 6), all data up until the visit before the group status is confirmed is used in the prediction. The final two columns present the results of prediction using LDA and QDA based on baseline characteristics and using no longitudinal information.

To illustrate our allocation scheme outlined in Section 4.1 and to help to interpret the parameter estimates in context of discrimination, we present the longitudinal data of two patients in Table 4, one patient who achieved remission and another who had refractory epilepsy. We present for each patient the time of their clinic visits and their longitudinal information gathered at each visit. First consider the refractory patient, Patient (a). At his first four appointments, although he has had many seizures and in some cases experienced adverse events, his probability of being in the refractory group does not yet rise above 0.74 (the cutoff determined to be optimal in Table 5). Up until this point, he would not be predicted as refractory and would remain under observation. Only at the fifth visit does this probability rise above the cutoff of 0.74 and at this point the marginal prediction method allocates him to the refractory group. For this particular patient, this turned out to be the correct prediction as can be seen by viewing his further clinic visits. By considering his baseline characteristics with the estimated model parameters in Table 3, we see that patients with generalized epilepsy have increased likelihood of experiencing seizures (and in fact many seizures) even if the patient would ultimately achieve remission. This is one reason why Patient (a) initially has low probability of being in the refractory group despite experiencing seizures. At these early time points, we are not yet sufficiently confident that we can predict he will be refractory. However, we are still able to accurately classify him after 833 days (approximately two years and three months) which is considerably earlier than waiting five years to determine their status.

In contrast, Patient (b) ultimately achieves remission. He has initially low probabilities of being refractory due to having no seizures. When he does experience seizures, his probability of being refractory increases to 0.23 but is still well below the required cutoff of 0.74. As this patient experiences no further seizures, his probability of being refractory drops again and at the visit prior to remission being confirmed he is correctly classified as remission. This is confirmed to be correct at his visit when *t* = 862 days since it is observed that he has had at least 12 months without experiencing seizures.

The allocation scheme has been specifically designed to identify refractory patients. We have set up a scheme whereby as soon as a patient is classified as refractory; we stop predicting for this patient and investigate alternative treatment options. Questions may arise in these kind of settings as to how long one must wait to be confident of the prediction. We have shown that by observing a patient until their probability of being refractory is greater than 0.74 then over 90% of remission and refractory patients are correctly identified.

A further significant finding in the example is the gain in lead time by using the dynamic approach. We define the lead time as the average time, before clinical classification can be confirmed, at which our method can correctly predict a patient as belonging to the refractory group. The corresponding prediction time is the average time since diagnosis at which patients are correctly identified as belonging to the refractory group. We emphasize that these two measures are calculated using those patients who were truly refractory and also predicted to be refractory by the model. The lead times shown in Table 5 consider those patients who are truly in the refractory group and are predicted to be in the refractory group. For the dynamic marginal prediction method, the lead time is 651 days. This means that we can identify those patients who will not achieve remission from seizures almost two years before they are clinically observed as such on average. This is a good time gain, allowing clinicians to consider other forms of treatment, so that patients do not have to endure the adverse side effects of unsuitable treatments.

**Figure 3.**
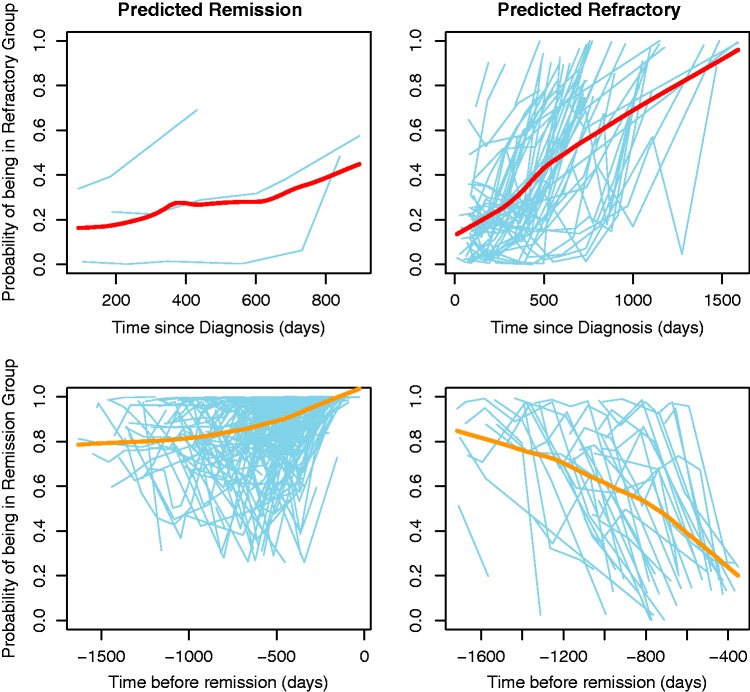
Changes of marginal group membership probabilities over time. The profiles are from one test set of 30% of patients. Their probabilities are calculated using the model developed on the remaining 70% of patients. The top row shows those patients whose true status is refractory whilst the bottom row shows the true remission patients. The left hand panels show all patients who are classed as remission within five years. The right panels show the patients who are predicted as refractory (up until the point at which they are classified as refractory).

We now further explore the dynamic LoDA scheme. We chose one of the 100 splits of data into training and test sets. We chose a split such that the sensitivity and specificity were close to the average sensitivities and specificities over the 100 splits. Using a cutoff of 0.74 (determined to be optimal for the marginal prediction, see Table 5), patients were predicted as either refractory or remission using our proposed allocation scheme. The profiles of patients assigned to each of the remission and refractory groups based on a marginal prediction scheme are shown in Figure 3. Refractory patients that are misclassified as remission cases (three patients, top row) have low probabilities. This was due to infrequent seizures and generally low numbers of seizures.

Most of the patients who are predicted correctly as refractory have high probabilities almost immediately of being in the refractory group. These are identified early which is consistent with the good lead times achieved, as shown in Table 5. Some of the patients who are truly refractory but were classed as remission could be correctly classified by lowering the cutoff (e.g., to 0.5). However, this would be at the cost of increasing the misclassification rate of remission cases.

In the bottom row of Figure 3, the true remission cases are shown. Most of the patients correctly identified as being in the remission group have high probabilities of being in the remission group very early on. Those patients who are wrongly predicted as refractory are generally those who have been observed for longer and hence taken longer to achieve remission. Such patients may initially have high numbers of seizures and so have initially high probabilities of being in the refractory group. A limitation of our allocation scheme is that these patients would be classed as refractory and then prediction would stop for these patients. It is possible that if they were observed for longer, their probabilities of being in the remission group would increase. This is a limitation with any classification scheme where an intervention is planned following a positive result.

### 4.4 Which longitudinal biomarkers to use

The longitudinal biomarkers we consider in our model are clearly correlated. In particular, there is a high degree of correlation between the binary biomarker describing whether a patient experienced seizures or not and the continuous biomarker describing how many seizures they experienced. The three markers were chosen to illustrate different types of longitudinal marker. In this section, we investigate what effect adding or removing any of the three biomarkers has on the predictive accuracy. Under the same procedure of splitting the data into training and test sets 100 times and averaging the predictive accuracy measures, we compared each of the combinations of the three longitudinal biomarkers considered in this paper and present the results in Table 6.

**Table 6.** Comparison of possible models under the marginal prediction scheme based on averages of 100 splits of the data into training and test sets.

	Cutoff	Sensitivity	Specificity	PCC	AUC	PPV	NPV	Mean lead	Mean prediction
								Time (days)	Time (days)
*Y*_1_	0.61	0.94	0.94	0.94	0.98	0.64	0.99	502	1041
*Y*_2_	0.43	0.89	0.87	0.87	0.94	0.45	0.99	860	666
*Y*_3_	0.13	0.71	0.69	0.70	0.78	0.22	0.95	1001	535
$Y_{1}+Y_{2}$	0.75	0.93	0.92	0.92	0.97	0.57	0.99	656	871
$Y_{1}+Y_{3}$	0.54	0.94	0.92	0.92	0.97	0.58	0.99	593	952
$Y_{2}+Y_{3}$	0.45	0.90	0.89	0.89	0.95	0.49	0.99	834	692
$Y_{1}+Y_{2}+Y_{3}$	0.72	0.94	0.92	0.92	0.97	0.58	0.99	659	869

PCC: probability of correct classification; AUC: area under curve; PPV: positive predictive value; NPV: negative predictive value.

*Y*_1_ denotes whether a patient experienced seizures or not since the previous visit, *Y*_2_ describes the total number of seizures experienced since the previous visit under the transformation log(1+totalseizures) and *Y*_3_ describes the number of adverse events experienced since the previous visit. The optimal cutoffs for each model were determined by ROC analysis by selecting the top left most point of the ROC curve.

The predictive accuracy of the univariate model involving the binary variable, *Seizures*, is comparable to the predictive accuracy of the trivariate model. However, with the trivariate model, patients can be correctly identified approximately five months earlier. So in our example, considering multiple markers does not improve the predictive accuracy, but does add information that allows prediction of refractory patients to be made earlier than by simply considering a single biomarker.

### 4.5 Benefits of dynamic LoDA

Dynamic LoDA has received increased attention in recent years in the statistical literature. It has become very desirable to have methods of prediction that can be updated at each time point. The alternative to this is to wait until all the data are gathered and then make a prediction. In our application, this would involve waiting for almost five years in order to determine patients’ status. Obviously, in this scenario, there would be no need for classification methods since we would simply have observed which group patients belong to. We would have no misclassification but at the cost of giving some patients ineffective treatment for potentially five years.

By contrast, with our dynamic allocation scheme, the risk is that a patient could have been wrongly classified as refractory, when if followed up a bit longer they would have been classified as remission.

**Figure 4.**
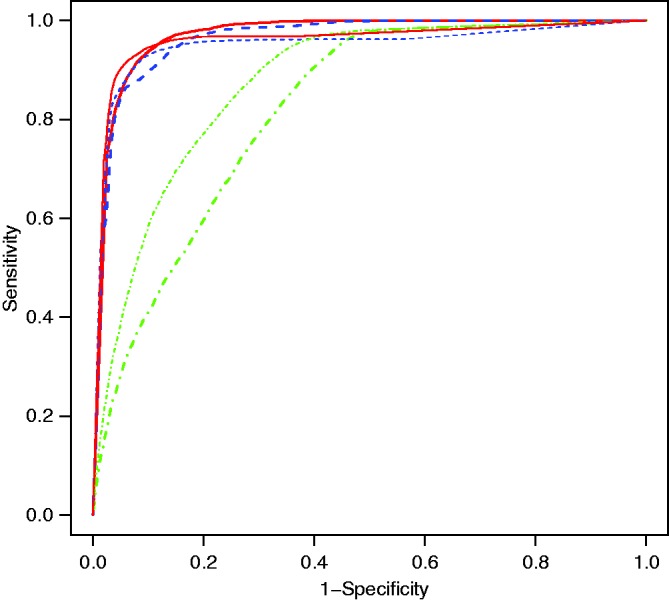
Receiver Operating Characteristic curves of the prediction using the marginal (solid red), conditional (dashed blue) and random effects (dot dashed green) prediction methods. The thick lines represent the dynamic allocations whilst the thin lines represent the use of the full data.

It is commonly thought that observing a patient for longer leads to increased information and so increased accuracy in prediction. We explored this in our example, by comparing the prediction results in the first three columns of Table 5 with those obtained by using all information gathered on a patient up until the visit before their status was confirmed (columns 4 to 6 of Table 5). In this setting, we use all available longitudinal information for each patient. The benefit of waiting until all information is gathered is a small increase in the PCC and specificity, while no benefit is observed in sensitivity or AUC for the marginal prediction (Table 5 and Figure 4). The most evident advantage of the dynamic LoDA over the use of the full data is the significant difference in lead times and prediction times. By waiting for all the data to be collected, patients would have to wait more than two years extra to be classified, whilst only making a minimal gain in predictive accuracy.

At the other extreme, an alternative would be to simply predict a patients’ group membership at diagnosis, based on various baseline characteristics and take no account of accumulating longitudinal information. We examined this possibility using traditional linear and quadratic discriminant analysis methods (LDA and QDA see Chapter 4 in Hastie et al.^10^). These results, also based on 100 splits of the data into training and test sets, are presented in the final two columns of Table 5. Although reasonably accurate prediction can be made at diagnosis, significant improvements in predictive accuracy can be obtained by updating predictions as new information becomes available for each patient.

In this section, we show that there is merit in considering how a patient’s clinical data change over time during observation and updating the prediction of their five-year status each time new information is available. In addition, we have shown that allocating a patient to the refractory group as soon as their probability of being in the refractory group rises above a cutoff (as opposed to observing the patient for five years) does not decrease the predictive accuracy and allows refractory patients to be identified much earlier on.

## 5 Discussion

In this paper, we propose a time-dependent discriminant analysis approach that allows for the inclusion of multiple longitudinal biomarkers of various types. Binary, Poisson and continuous longitudinal markers can be included within a MGLMM. An implementation of the methods described in this paper has now been added to the package mixAK^30^ of the R software.^31^

The longitudinal profiles of considered biomarkers are described using GLMMs. We have allowed for extra flexibility through the inclusion of a mixture distribution of the random effects. These random effects capture the correlation between markers and between observations of a particular marker.

In the clinical application with SANAD data, the inclusion of a normal mixture for the random effects distribution showed only a mild impact in classification accuracy. Nevertheless, the impact can in general be much more considerable. An example of such situation is when one of the groups is characterized by subdivisions of different longitudinal behaviour of the considered markers. This subdivision might not be of interest for classification, nevertheless, if properly taken into account, e.g., by assuming a mixture distribution for random effects, it may considerably improve the classification accuracy. Moreover, since mixtures are in general considered as a suitable semi-parametric model for unknown distributions, they are more able to adapt to model misspecification, and so should be considered as a way of limiting the effect of model misspecification. In addition to reducing the chances of model misspecification, including mixtures may in some cases improve the fit of the model by reducing measures such as the PED. Checking improvement of model fit and in predictive accuracy will determine if this methodology will be a useful tool in any particular example.

In our context, the SANAD database which has more than 1700 patients allowed us to fit reasonably complex models containing three longitudinal markers and six covariates for each of them. We must point out that if very small sample sizes were available then more simple models may need to be considered. Some insight into how large sample is needed to fit models of given complexity can be gained from Komárek and Komárková^29^ who present results of a simulation study towards properties of the estimators of parameters of the MGLMM that is behind our LoDA procedure.

One of the limitations in our application is that once a patient has achieved remission, the follow-up data after achieving remission are discarded. This has a direct effect on the length of follow up for some patients in the remission group, although conceptually one could argue that only the profile of the longitudinal biomarkers before achieving remission are of interest. A possible consequence of this is that the longitudinal profile of remission patients at late time points may be less accurately estimated since fewer remission patients are observed for that long. The limitation from a clinical point of view is that relapse in patients with epilepsy after achieving remission is not considered here.

Using longitudinal information along with dynamic LoDA schemes has been seen to give good classification results, yielding good prediction accuracy. In addition, we are able to make predictions about patients substantially earlier than is currently possible showing the potential benefits of such an approach.

With our dynamic classification scheme used for the SANAD application, we dynamically update the allocation probabilities as new longitudinal information arrives, nevertheless, prediction of the group pertinence is performed for each patient only once. Indeed, each patient remains unclassified till either his allocation probability of being refractory exceeds the cutoff value or those allocation probabilities remain below the cutoff value for a predefined period of time (five years in our case). Consequently, standard accuracy measures (such as AUC, sensitivity, specificity, etc.) were applied to evaluate discrimination ability of our procedure. Alternatively, at each visit, we could have used the allocation probabilities and predicted the group allocation. This would then also possibly change dynamically over time and different approach would have to be taken to evaluate a discriminant ability of the LoDA procedure. To this end, one could adopt an extended definition of sensitivity, specificity and dynamic AUC as proposed by Heagerty and Zheng^36^ in context of survival analysis and then further generalized in different contexts.^37^ Nevertheless, since our main focus here was ultimately on identifying refractory patients at any point within the five-year period, we do not pursue this idea further in this paper.

We compared three approaches to prediction, namely marginal, conditional and random effects prediction and found that for our application both the marginal and the conditional approaches gave good prediction, with the marginal approach most often being the best. The random effects prediction was less accurate for the SANAD data.

We believe our methods could be used in a wide variety of applications. They allow for irregularly collected data, multivariate longitudinal data and can incorporate data of different types. Classification into prognostic groups based on biomarker evolution is an increasingly important aspect of clinical practice and the approach proposed here has the flexibility to be used with many different clinical biomarkers, increasing the options available to researchers. A useful extension to this work would be to allow for discrimination using genuine categorical or ordinal biomarkers. To this end, suitable regression models suggested recently in the literature^38,39^ for such outcomes could be considered.

In this paper, we present an example where patients are classified into one of two groups. However, the methods here presented are applicable for classification into three or more groups as, for example, in applications where the aim is to classify patients into various stages of cancer (as opposed to simply cancer against cancer free patients) giving wider applicability to the methods proposed.
